# MicroRNA-143 sensitizes acute myeloid leukemia cells to cytarabine via targeting ATG7- and ATG2B-dependent autophagy

**DOI:** 10.18632/aging.103614

**Published:** 2020-10-19

**Authors:** Hao Zhang, Jianmin Kang, Ling Liu, Lulu Chen, Saisai Ren, Yanling Tao

**Affiliations:** 1 Department of Hematology, Affiliated Hospital of Jining Medical University, Jining 272029, Shandong Province, China; 2 Department of Hematology, The Second Hospital of Shanxi Medical University, Taiyuan 030001, China; 3 Graduate School, Jining Medical University, Jining 272000, Shandong Province, China; 4 Department of Pediatric Hematology, Affiliated Hospital of Jining Medical University, Jining 272029, Shandong Province, China

**Keywords:** microRNA-143, acute myeloid leukemia, cytarabine, autophagy, ATG7

## Abstract

Targeting autophagy holds promise to enhance chemosensitivity in acute myeloid leukemia (AML). MicroRNA-143 (miR-143) has been found to suppress autophagy, however, it is not clear whether miR-143 augments cytarabine cytotoxicity in AML. Here, we report that cytarabine treatment reduces miR-143 expression in AML cell lines and primary AML cells. Moreover, ectopic expression of miR-143 further decreases cell viability in cytarabine-treated AML cells. By contrast, miR-143 knockdown inhibits cytarabine-induced cytotoxicity, together indicating a role of miR-143 in enhancing cytarabine sensitivity in AML. Subsequently, we show that miR-143 inhibits autophagy in cytarabine-treated AML cells by directly targeting autophagy-related proteins (ATG), ATG7 and ATG2B, two critical known components of autophagic machinery. More importantly, autophagy reconstructed via co-expression of ATG7 and ATG2B substantially attenuates miR-143-enhanced cytotoxicity, which is associated with suppression of caspase-dependent apoptotic pathway. Overall, this study demonstrates that targeting ATG7 and ATG2B-dependent autophagy is a critical mechanism by which miR-143 sensitizes AML to cytarabine, implicating it as a potential therapeutic target in AML treatment.

## INTRODUCTION

Acute myeloid leukemia (AML) is a rapidly progressing malignancy characterized by immature differentiation and abnormal proliferation of hematopoietic precursors in bone marrow and blood [1, 2]. Although AML accounts for only 20% of pediatric leukemia cases, it is disproportionately responsible for more than 30% of mortality in suffering children [3, 4]. In current therapies for AML, cytarabine, a DNA nucleoside analog that disrupts DNA synthesis, serves as an essential and effective cytotoxic agent in both primary and salvage chemotherapy regimens [5]. Despite a high early remission rate, most AML cases frequently relapse and gain resistance against cytarabine, leading patients to succumb to the disease [6]. The 5-year survival rate for AML patients is only about 30%, which is even worse among elderly patients [7]. Due to this major clinical challenge, it is urgent to understand the underlying molecular basis and identify therapeutic targets that could overcome cytarabine resistance.

In the last decade, considerable attention has been paid to study the role of autophagy in chemoresistance [8]. Autophagy is an evolutionarily conserved catabolic pathway constituting a cellular quality control mechanism. It promotes bulk degradation of intracellular substrates in lysosomes, such as aggregated or misfolded proteins and impaired organelles [9]. In addition to homeostatic functions, autophagy has also been shown to increase cytotoxic drug resistance during chemotherapy that helps cancer cell to survive [10]. For instance, inhibition of autophagy was found to sensitize AML cells to cytarabine treatment in vitro [11–13]. However, the mechanisms for autophagy-mediated cytarabine resistance are still largely undefined and the prospect of directly targeting autophagy remains poor.

MicroRNAs (miRNAs) are defined as small non-coding RNAs with 19 to 25 nucleotides in length. miRNAs can bind to the 3’ untranslated region (3’-UTR) of target mRNAs, resulting in translational repression or gene silencing [14]. Many studies have demonstrated that miRNAs play important roles in a variety of vital biological processes, such as proliferation, differentiation, apoptosis, autophagy and aging [15]. Moreover, miRNAs also influence malignant transformation and chemoresistance in AML [16, 17]. Recently, miR-143 was identified as a relevant prognostic and therapeutic factor in AML therapy [18]. Furthermore, miR-143 inhibits autophagy in non-small cell lung cancer cells and gastric cancer cells to improve chemoresistance towards quercetin [19, 20]. These reports intrigued us to ask whether and how miR-143 enhances cytarabine cytotoxicity in AML cells. In this study, we show that miR-143 expression in AML cells is decreased upon treatment with cytarabine. Further, using overexpression and knockdown strategies, we demonstrate that miR-143 enhances cytarabine cytotoxicity in AML cells by suppressing autophagy through targeting ATG7 and ATG2B.

## RESULTS

### miR-143 expression is decreased in cytarabine-treated AML cells

**Figure 1:**
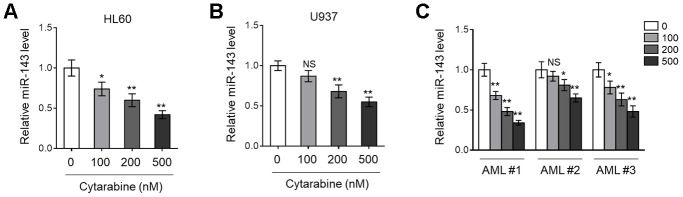
**Cytarabine treatment decreases miR-143 expression in AML cells.** (**A**, **B**) Human AML cell line HL60 (**A**) and U937 (**B**) were treated with increasing concentrations of cytarabine as indicated for 24 h. The expression level of miR-143 was determined by RT-qPCR analysis. The house-keeping gene *ACTB* was used as a reference control. The results are expressed as relative to vehicle group. (**C**) Three lines of primary AML cells from newly diagnosed patients (named as AML #1, AML #2, AML #3) were individually treated as in (**A**, **B**). The analysis of expression level of miR-143 was conducted as in (**A**, **B**). Each column represents the value from 5 replicates. All data are mean ± SD from three independent experiments. Data between two groups were compared using Student *t*-test. **, P<0.01; *, P<0.05; NS, not significant, versus 0 nM group in each cell line.

Previous studies have found that miR-143 is frequently downregulated in various types of cancer, including hematopoietic malignancies [24, 25]. More recently, miR-143 expression has been shown to predict outcome of AML patients [18]. To explore whether miR-143 is associated with cytarabine cytotoxicity in AML cells, we first monitored its expression in cytarabine-treated human AML cell lines, HL60 and U937, using RT-qPCR analysis. The results showed that miR-143 expression in both HL60 (Figure 1A) and U937 (Figure 1B) cells was decreased by cytarabine treatment in a dose-dependent manner. Similar tendency was found in a time-dependent manner (data not shown). To establish a closer relevance to the clinical settings, AML cells from 3 newly diagnosed patients were collected, and further expanded and treated with cytarabine *in vitro*. Astonishingly, similar to that found in HL60 and U937 cells (Figure 1A, 1B), miR-143 expression was also dose-dependently downregulated in these primary AML cells (Figure 1C). Hence, the results from both AML cell lines and clinical specimens suggest that miR-143 responds to cytarabine treatment by downregulation.

### miR-143 enhances cytarabine-induced cytotoxicity in AML cells

**Figure 2:**
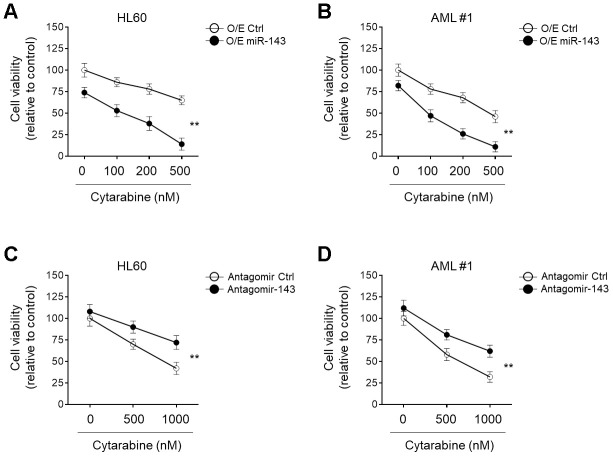
**miR-143 enhances cytotoxicity of cytarabine in AML cells.** (**A**, **B**) HL60 (**A**) and AML #1 (**B**) cells were transfected with 100 nM miR-143 mimic (O/E miR-143) or 100 nM non-target mimic control (O/E Ctrl) for 48 h, and then treated with increasing concentrations of cytarabine as indicated for 24 h. Cell viability was analyzed by MTT assay. The results are expressed as relative to vehicle group (%). (**C**, **D**) HL60 (**C**) and AML #1 (**D**) cells were transfected with 100 nM antagomir of miR-30a (Antagomir-143) or 100 nM non-target antagomir (Antagomir Ctrl) for 48 h, and then treated with increasing concentrations of cytarabine as indicated for 24 h. Cell viability was analyzed and expressed as in (**A**–**B**). Each symbol represents the value from 5 replicates. Data were compared using two-way ANOVA with a post hoc Tukey’s test. **, P<0.01.

miRNA signatures are associated with chemosensitivity in tumors, including AML [26–28]. The downregulation of miR-143 in cytarabine-treated AML cells led us to evaluate its possible role in cytarabine cytotoxicity. To explore this, miR-143 was ectopically overexpressed in HL60 cells and then cells were treated with cytarabine. Cells viabilities were determined using MTT assays. The result showed that in comparison to control samples, miR-143 overexpression resulted in further decreased cell viability upon cytarabine treatment (Figure 2A), indicating an enhanced cytarabine cytotoxicity in HL60 cells upon miR-143 overexpression. Additionally, similar results were also obtained when primary AML cells were investigated (Figure 2B). On the other hand, when miR-143 was knocked down by transfection of an antagomir, the survival rate of cytarabine-treated HL60 cells (Figure 2C) and primary AML cells (Figure 2D) was profoundly improved. In addition to MTT assays, similar results were obtained using CCK-8 experiment (Supplementary Figure 1A, 1B). And all the above phenomena were also reproducible in a time-dependent manner (data not shown). Taken together, these lines of evidence suggest that miR-143 functions to increase cytarabine cytotoxicity in AML cells, at least *in vitro*.

### miR-143 inhibits autophagy in cytarabine-treated HL60 cells

**Figure 3:**
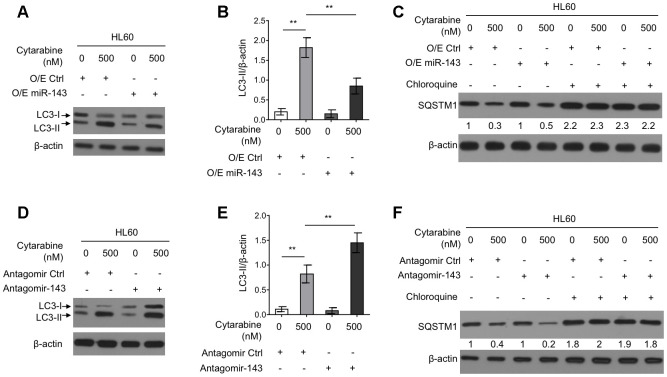
**miR-143 inhibits cytarabine-induced autophagy in HL60 cells.** (**A**, **B**) HL60 cells were transfected with 100 nM O/E miR-143 or 100 nM O/E Ctrl for 48 h, and then treated with or without 500 nM cytarabine for 24 h. The protein expression of LC-3 was measured by immunoblotting. β-actin was used as a loading control. The representative images (**A**) and statistical analysis of LC3-II/β-actin (**B**) are shown. (**C**) HL60 cells were transfected as in (**A**) and treated with cytarabine in the presence or absence of 30 μM chloroquine. The expression of SQSTM1 was analyzed by immunoblotting. (**D**, **E**) HL60 cells were transfected with 100 nM Antagomir-143 or 100 nM Antagomir Ctrl for 48 h, and then treated with or without 500 nM cytarabine for 24 h. The protein expression of LC-3 (**D**) and statistical analysis of LC3-II/β-actin (**E**) were conducted as in (**A**, **B**). (**F**) HL60 cells were transfected as in (**D**) and treated with cytarabine in the presence or absence of 30 μM chloroquine. The expression of SQSTM1 was analyzed by immunoblotting. All data were from 3 independent experiments and expressed as mean ± SD. Data were compared using Student *t*-test. **, P<0.01.

Autophagy is an important intracellular physiological process that influences cytarabine chemosensitivity in leukemia cells [8]. It has also been reported that cytarabine treatment induces autophagy in AML cells [13, 29]. Further, some literatures have shown that miR-143 inhibits autophagy in the non-small cell lung cancer H1299 cells and gastric cancer cells [19, 20]. However, whether miR-143 modulates autophagy and cytarabine chemosensitivity in HL60 cells is unknown. To gain mechanistic insight into the enhanced cytarabine cytotoxicity by miR-143, we assessed autophagy level in cytarabine-treated HL60 cell by manipulating miR-143 expression levels. Western blotting analysis showed that cytarabine treatment induced the microtubule-associated protein1 light chain 3 (MAP1LC3)-II (LC3-II) turnover, a widely-used prominent indicator of autophagy [30], which was, however, suppressed by miR-143 overexpression (Figure 3A, 3B). Furthermore, miR-143 overexpression inhibited cytarabine-induced degradation of an autophagic substrate sequestosome 1 (SQSTM1), though this phenotype was abolished when an autophagic inhibitor chloroquine was present (Figure 3C). In other set of experiments, miR-143 knockdown led to elevated autophagy levels in cytarabine-treated HL-60 cells, which was evidenced by elevated LC3-II turnover (Figure 3D, 3E) along with increased SQSTM1 degradation (Figure 3F). Overall, these results indicate that miR-143 inhibits cytarabine-induced autophagy in HL60 cells, and this effect may be associated with miR-143-promoted cytarabine cytotoxicity.

### miR-143 directly targets ATG7 and ATG2B in HL60 cells

**Figure 4:**
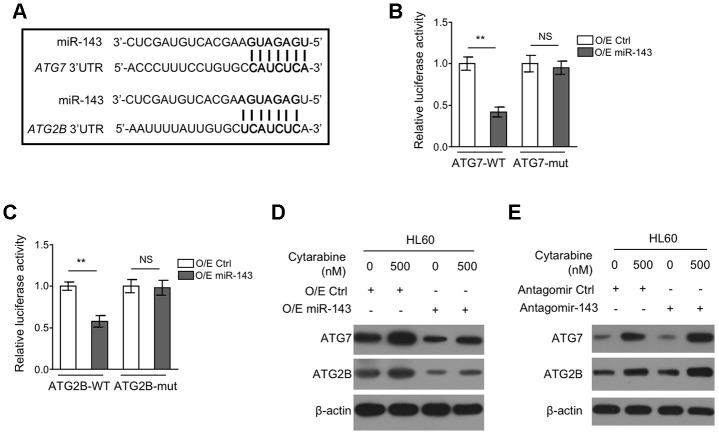
**miR-143 decreases expression of ATG7 and ATG2B by directly targeting in HL60 cells.** (**A**) Schematic illustration of the complementary sequence between miR-143 and the 3’-UTRs of ATG7 and ATG2B mRNAs. This information is provided by the computational and bioinformatics-based approach using TargetScan. (**B**) HEK293 cells were co-transfected pMIR-LUC-3’-UTR-ATG7-wt (ATG7-wt) or pMIR-LUC-3’-UTR-ATG7-mut (ATG7-mu) with 100 nM O/E miR-143 or 100 nM O/E Ctrl for 48 h, and then luciferase activity was measured. The results relative to O/E Ctrl group are shown. (**C**) HEK293 cells were co-transfected pMIR-LUC-3’-UTR-ATG2B-wt (ATG2B-wt) or pMIR-LUC-3’-UTR-ATG2B-mut (ATG2B-mu) with 100 nM O/E miR-143 or 100 nM O/E Ctrl for 48 h, and then luciferase activity was measured. The results relative to O/E Ctrl group are shown. (**D**) HL60 cells were transfected with 100 nM O/E miR-143 or 100 nM O/E Ctrl for 48 h, and then treated with or without 500 nM cytarabine for 24 h. The protein expression of ATG7 and ATG2B was measured by immunoblotting. β-actin was used as a loading control. (**E**) HL60 cells were transfected with 100 nM Antagomir-143 or 100 nM Antagomir Ctrl for 48 h, and then treated with or without 500 nM cytarabine for 24 h. The protein expression of ATG7 and ATG2B was analyzed as in (**D**). All data were from 3 independent experiments and expressed as mean ± SD. Data were compared using Student *t*-test. **, P<0.01; NS, not significant.

To understand how miR-143 inhibits autophagy, we explored its potential autophagic targets using target prediction algorithms including TargetScanHuman version 7.2 and miRbase, and the putative binding sites of miR-143 within the 3’UTR of autophagy-related protein 7 (ATG7) and ATG2B mRNA were predicted (Figure 4A), which are two critical components for autophagy formation [31, 32]. To confirm their interaction with miR-143, a dual luciferase assay using luciferase reporter vector expressing wild-type or mutant 3’-UTR of ATG7 and ATG2B was introduced in miR-143-overexpressing HL-60 cells. The results showed that in comparison to overexpression control, miR-143 overexpression pronouncedly inhibited the luciferase activity of both ATG7-WT (Figure 4B) and ATG2B-WT (Figure 4C) constructs. Whereas, similar effects were not seen when miR-143 was co-expressed with mutant construct ATG7-mut (Figure 4B) or ATG2B-mut (Figure 4C), showing that miR-143 directly interacts with the 3’-UTR of ATG7 and ATG2B mRNA. Coinciding with its inducible effect on autophagy in HL-60 cells, cytarabine treatment increased protein expression of ATG7 and ATG2B (Figure 4D, 4E). Furthermore, overexpression of miR-143 reduced expression of ATG7 and ATG2B (Figure 4D), and oppositely, miR-143 knockdown increased their expression (Figure 4E). Overall, these findings confirm that miR-143 reduces the expression of ATG7 and ATG2B in cytarabine-treated HL-60 cells, which may be responsible for the aforementioned autophagy inhibition (Figure 3).

### Autophagy restoration through ectopic co-expression of ATG7 and ATG2B diminishes miR-143-enhanced cytarabine cytotoxicity in HL60 cells

**Figure 5:**
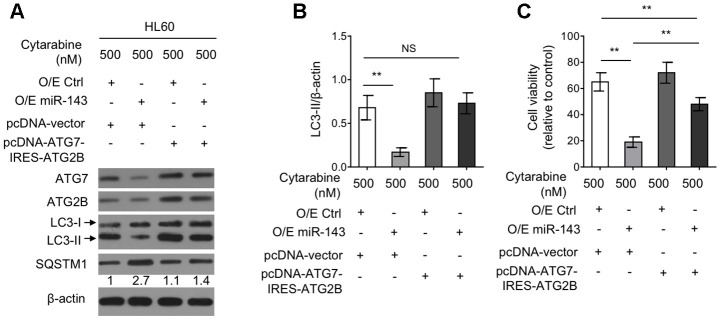
**Ectopic co-expression of ATG7 and ATG2B restores autophagy and diminishes miR-143-enhanced cytarabine cytotoxicity in HL60 cells.** (**A**, **B**) HL60 cells were co-transfected 100 nM O/E miR-143 or 100 nM O/E Ctrl with pcDNA-vector or pcDNA-ATG7-IRES-ATG2B for 48 h in the presence of 500 nM cytarabine. The protein expression of ATG7, ATG2B, and LC-3 was measured by immunoblotting. β-actin was used as a loading control. The representative images (**A**) and statistical analysis of LC3-II/LC3-I (**B**) are shown. (**C**) HL60 cells were treated as in (**A**, **B**). Cell viability was analyzed by MTT assay. The results are expressed as relative to vehicle group (%). In (**A**, **B**), data are representative of 3 independent experiments. In (**C**), each symbol represents the value from 5 replicates. Data are mean ± SD and compared using Student *t*-test. **, P<0.01; NS, not significant.

To elucidate the functional role of reduced expression of ATG7 and ATG2B in miR-143-suppressed autophagy as well as in miR-143-promoted cytarabine cytotoxicity, a tandem vector capable of simultaneously co-expressing ATG7 and ATG2B (pcDNA-ATG7-IRES-ATG2B) was transfected into cytarabine-treated HL-60 cells overexpressed with miR-143. The results from western blotting analysis demonstrated that along with the recovered expression of ATG7 and ATG2B by transfection of pcDNA-ATG7-IRES-ATG2B construct, the inhibited autophagy by miR-143 overexpression was restored to the level in overexpression control group, as evidenced by LC3-II turnover (Figure 5A, 5B), describing that miR-143 inhibits autophagy through decreasing the expression of ATG7 and ATG2B under this condition. Also, autophagy restoration drastically increased cell viability in miR-143-overexpressing HL-60 cells treated with cytarabine, although did not completely reach to that of overexpression control group (Figure 5C). Nonetheless, these evidence together suggest that miR-143-mediated autophagy inhibition via suppressing expression of ATG7 and ATG2B plays a critical role in enhancing cytarabine cytotoxicity in AML cells.

### Decreases in ATG7 and ATG2B contribute to miR-143-promoted caspase-dependent apoptosis in cytarabine-treated HL60 cells

**Figure 6:**
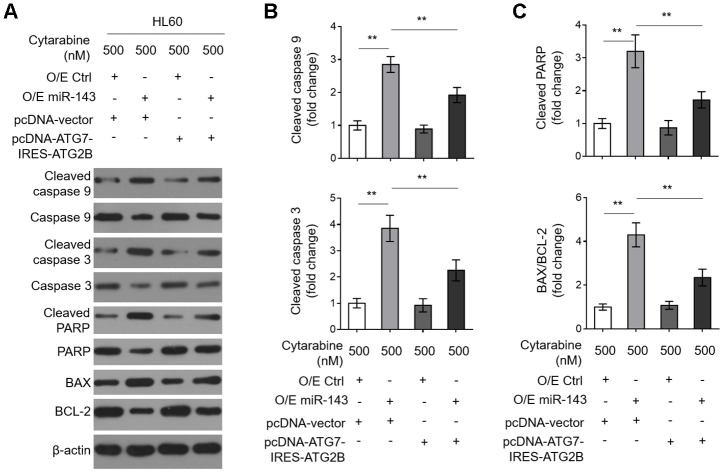
**Decreased expression in ATG7 and ATG2B contributes to miR-143-promoted apoptosis in cytarabine-treated HL60 cells.** (**A**–**C**) HL60 cells were treated as in Figure 5A. The expression of indicated protein targets was measured by immunoblotting. β-actin was used as a loading control. The representative images (**A**) and statistical analysis of the fold change of key protein targets (**B**, **C**) are shown. All data are representative of 3 independent experiments. Data are mean ± SD and compared using Student *t*-test. **, P<0.01.

**Figure 7:**
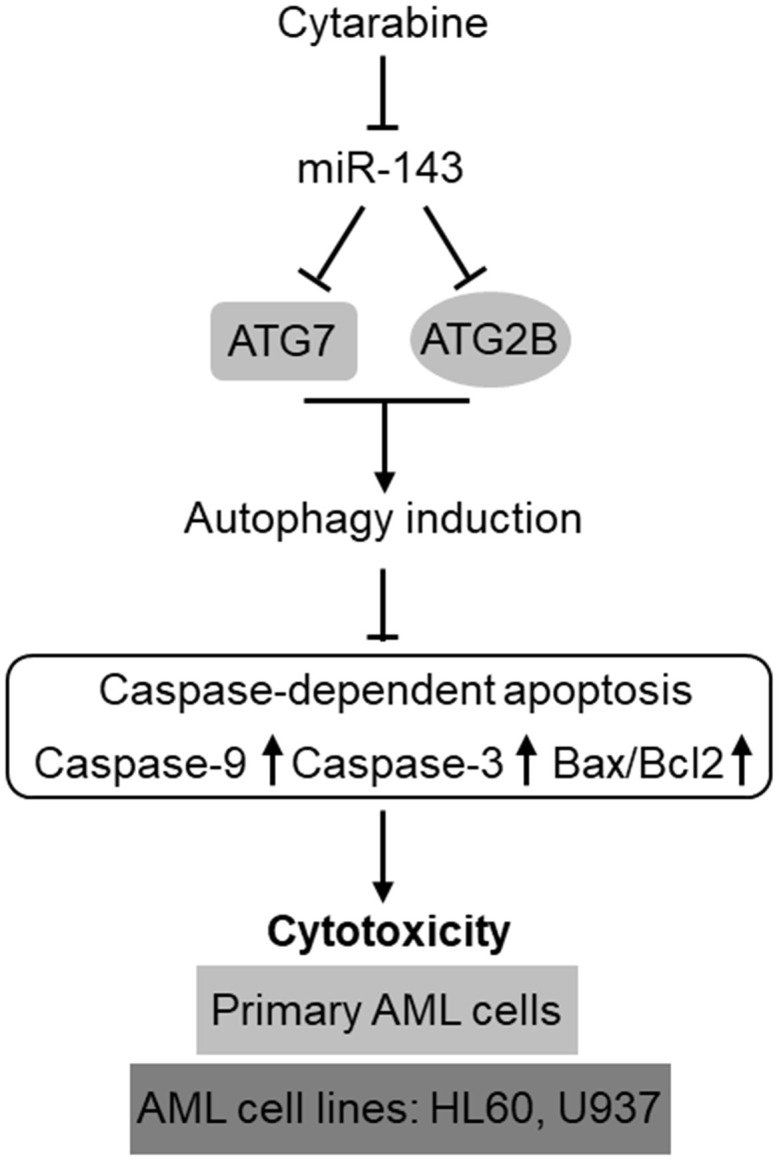
**Schematic description of the role and mechanism by which miR-143 influences cytarabine activity against AML.** MiR-143 functions to inhibit autophagy induction via targeting ATG7 and ATG2B, whereby erasing the inhibitory role of autophagy in cytarabine-induced caspase-dependent apoptosis and cytotoxicity in AML cells, including primary AML cells and human AML cells lines, HL60 and U937. However, the expression of miR-143 in AML cells is downregulated by cytarabine treatment, thus compromising the cytarabine cytotoxicity against AML cells.

To learn further about the intrinsic mechanism by which miR-143 enhances cytarabine cytotoxicity in AML cells, we investigated its effect on cytarabine-induced caspase-dependent apoptosis, which is fundamental for cytarabine cytotoxicity [33]. We found that miR-143 overexpression sharply increased expression of cleaved caspase 9 and cleaved caspase 3 (Figure 6A, 6B). It also resulted in elevated cleaved poly ADP-ribose polymerase (PARP) and BCL2 associated x (BAX)/B cell CLL/lymphoma-2 (BCL-2) in cytarabine-treated HL-60 cells (Figure 6A and 6C), clearly indicating that miR-143 overexpression promotes caspase-dependent apoptosis. Moreover, consistent with above findings, miR-143 overexpression also led to upregulation of cleaved PARP (Figure 6A) and cytosol release of cytochrome c (Cyto C) (Supplementary Figure 2A, 2B), strengthening the notion that miR-143 causes promoted activation of mitochondrial apoptosis pathway in cytarabine-treated HL-60 cells. However, upon restored expression of ATG7 and ATG2B, the effects of miR-143 overexpression on mitochondrial- and caspase-dependent apoptosis were diminished (Figure 6A–6C and Supplementary Figure 2A, 2B), which is in agreement with the recovered cell viability (Figure 5C). In summary, this study suggests that miR-143 enhances cytarabine cytotoxicity in AML cells by downregulating the anti-apoptotic autophagy machinery targets ATG7 and ATG2B (Figure 7).

## DISCUSSION

The development of chemoresistance in AML cells to cytarabine-based therapy is a huge obstacle for improving the clinical outcome in AML patients [34]. The poor overall prognosis due to chemoresistance has made it as a pressing need to comprehensively delineate the underlying molecular mechanisms so as to develop effectively-targeted approaches to treat the relapsed/refractory AML [35, 36]. Emerging knowledge about the prognostic and functional role of miRNAs in AML has rendered them as promising targets in AML diagnosis, treatment, and reverse of chemoresistance [17, 37, 38]. In the present study, we identify miR-143 as a novel positive regulator of cytarabine-induced cytotoxicity in AML cells, in which the targeted anti-apoptotic machinery of autophagy represents a predominant mechanism, thus highlighting an important role of autophagy in mediating self-protection function against cytarabine-induced cytotoxicity and also exemplifying miR-143 as a possible druggable target among miRNAs that could be employed to enhance the effectiveness of chemotherapy for AML treatment (Figure 7).

miR-143 was previously reported to play a role during Fas-mediated apoptosis in human T-cell leukemia cells [39]. The overexpression of miR-143 was also found to inhibit the growth and induce apoptosis in human leukemia cells [40]. Further, studies have shown that the expression of miR-143 is downregulated in childhood-lineage acute lymphoblastic leukemia at initial diagnosis and in relapse phases [41], and an association of high miR-143 expression with a higher probability of survival exists in AML patients [18]. These literatures point to an anti-leukemic effect miR-143 may exhibit in AML cells. Prompted by these clues, we first sought the possible association between miR-143 and cytarabine-induced cytotoxicity in AML cells. Intriguingly, we found that in human AML cell line HL60 and U937 as well as in primary AML cells, cytarabine decreased miR-143 expression, suggesting that a reverse correlation exists between miR-143 expression and cytarabine treatment. It has been reported that the differentially expressed miRNAs in distinct stages of lymphopoiesis could be used as signatures for discriminating AML with different subtypes [42, 43], and that changes in the expression of several miRNAs may have functional relevance with drug resistance [44]. Whereas, for the majority of these aberrantly expressed miRNAs, the mechanisms that control their expression are largely unknown. In addition to the genetic mechanisms such as mutation, deletion, amplification, loss of heterozygosity and translocation, the epigenetic mechanisms including DNA methylation and histone code may be all possibly involved in [45]. In recent studies, the decreased expression of miR-143 was shown to be associated with the methylation modification on its gene promoter [46, 47]. In addition, cytarabine mediates genome wide methylation and alters gene expression in AML [48]. We thus guess that the decreased miR-143 expression by cytarabine treatment in AML cells may be attributed to the methylation-mediated epigenetic regulation. Following investigations are needed to address whether this is the case. More significantly, whether the expression change of miR-143 in response to cytarabine treatment is of prognostic significance merits further studies.

Subsequently, through gain-and loss-of-function studies, we demonstrated that miR-143 increased cytarabine cytotoxicity in AML cells *in vitro*, which might provide another layer evidence supporting its role as an anti-leukemic miRNA. We next discovered that miR-143 directly targeted ATG7 and ATG2B to inhibit the autophagic activity. Moreover, ectopic co-expression of ATG7 and ATG2B completely restored autophagy and markedly diminished the promotive effect of miR-143 on cytarabine cytotoxicity and caspase-dependent apoptosis in HL60 cells. These mechanistic findings not only prove that the targeted autophagy contributes greatly to miR-143 function in cytarabine cytotoxicity, but also suggest that other mechanisms may also play a role in this scenario. Previous studies have also reported that the deletion of ATG7 or ATG2B promotes caspase-dependent apoptosis in various human cells [49–52]. Further, the protective autophagy against caspase-dependent apoptosis is associated with the regulation of reactive oxygen species (ROS) [53, 54]. It is thus interesting to investigate whether ATG7- and ATG2B-mediated autophagy protects against caspase-dependent apoptosis of cytarabine-treated AML cells through modulating ROS. In addition, it’s been demonstrated that the impairment of the autophagy-lysosome pathway induces apoptosis mainly via excessive ER stress [55]. Further, the depletion of c-Myc impairs autophagy flux, thereby reducing phosphorylation of JNK1 and its downstream target anti-apoptotic molecule Bcl2, and knockdown of this proto-oncogenic transcriptional factor disrupts autophagosome formation [56, 57]. Besides, the autophagic cell death, in which the factors like the JNK signal, interferon-gamma, FAK and EGFR are deeply involved [55, 58–61]. We also proved that the inhibited Akt/mTOR signaling pathway is associated with miR-143-enhanced cytotoxicity (Supplementary Figure 3). These related clues may provide a possibility to link the miR-143-asscociated phenotypes we observed in cytarabine-treated AML cells.

It is also conceivable that except for those targets involved in autophagy machinery, according to the target prediction by algorithms, miR-143 could also inhibit the expression of other target genes. For instance, miR-143 targets ERK5 in AML cells [18], and the inhibition of ERK5 has been demonstrated to enhance cytarabine-induced apoptosis in AML cells [62]. Therefore, in addition to the targeted autophagy, the detailed mechanisms underlying miR-143-promoted cytarabine cytotoxicity remain to be excavated in the future, including the identification of the subordinate targets. Issues about how miR-143-targeted autophagy is connected to the activation of caspase-dependent apoptosis, and whether other types of cell death also emerge under this condition require extended investigations.

In summary, this study provides molecular basis demonstrating that miR-143 sensitizes AML cells to cytarabine treatment by suppressing anti-apoptotic autophagy through directly targeting ATG7 and ATG2B. Evidence obtaining from animal models is preferably needed to demonstrate whether miR-143 enhances cytarabine cytotoxicity *in vivo*.

## MATERIALS AND METHODS

### Patients and primary AML cell sampling

AML peripheral blood mononuclear cells (PBMCs) were obtained from three *de novo* pediatric patients in our hospital who were newly diagnosed with AML following the French-American-British criteria [21]. Mononuclear cells were isolated from the bone marrow samples using Ficoll density gradient centrifugation (GE Healthcare) according to the manufacturer’s instructions. The isolated cells were either stored at -80°C for future usage or directly cultured in RPMI 1640 medium supplemented with 10% fetal bovine serum (FBS), 2 mM L-glutamine and 1% penicillin-streptomycin solution in a humidified incubator with 5% CO_2_ at 37°C throughout the study and treated with cytarabine (Sigma-Aldrich). The study protocols were approved by the Ethics Committee of Affiliated Hospital of Jining Medical University. The informed consent was obtained from all patients prior to sampling.

### Cell lines, culture and treatment

The human AML cell lines U937 and HL60 were obtained from American Type Culture Collection (ATCC). These cell lines were cultured in RPMI 1640 medium conditions similar to those for primary AML cells. For cytarabine treatment, cells were seeded with a density of 5×10^5^ cells/ml one day before the experiment, and fresh medium was added together with different concentrations of cytarabine according to experimental design, with or without 30 μM chloroquine (Sigma-Aldrich).

### Cell transfection

HL60 cells were seeded into 6-well plates and allowed to reach approximate 60% confluence before transfection. A final concentration of 100 nM miR-143 mimics (O/E miR-143), control miRNA mimics (O/E Ctrl), antagomir of miR-143 (Antagomir-143), non-target antagomir (Antagomir Ctrl) were transfected with Lipofectamine 2000 (Invitrogen) according to the manufacturer's protocol. To restore expression of ATG7 and ATG2B, the construct of pcDNA-ATG7-IRES-ATG2B was established by cloning gene fragments of human ATG7 and ATG2B into the pcDNA vector (Genepharma) to achieve simultaneous double-overexpression of ATG7 and ATG2B. At 48 h or 72 h after transfection, HL60 cells were harvested for subsequent analyses.

### Cell viability determination

Cell viability was determined using the CellTiter Non-Radioactive Cell Proliferation Assay (MTT) (Promega) according to the manufacturer's instructions. Briefly, primary AML cells and HL-60 cells were plated into the 96-well plates with a density of 2×10^4^ cells. After transfection, cells were further incubated for 24 h in culture medium containing increasing concentrations of cytarabine (0, 100, 200 and 500 nM). Subsequently, MTT dye (20 μl per well) was added and further incubated for 4 h at 37°C. The formazan precipitate was dissolved using dimethyl sulfoxide (DMSO) (150 μl per well), and the absorbance was measured at 490 nm using an automatic microplate reader (Molecular Device). Each treatment was allocated with 5 replicates. The results were calculated according to a standard curve and expressed as relative to control treatment.

### Real-time quantitative PCR analysis

Total RNA was extracted from primary AML cells and cell lines using Trizol Reagent (Takara), and cDNA was synthesized using RevertAid First Strand cDNA Synthesis Kit (ThermoFisher Scientific). MicroRNA-143 expression was quantified using real-time quantitative PCR (RT-qPCR) with TaqMan microRNA assay (Applied Biosystems) on CFX96 PCR system (bio-rad). The housekeeping gene beta*-actin* (*ACTB*) was used for expression normalization of mRNAs. The specific sense and anti-sense primers used for amplifying human miR-143 were 5’- TGCTGGGTGCAGTGCTGCATCTCTGGTCAGTTGGGAGTCTGAGATGAAGCACTGTAGCTC-3’ and 5’-CCTGGAGCTACAGTGCTTCATCTCAGACTCCC AACTGACCAGAGATGCAGCACTGCACCC-3’ [22]. Notably, the efficiency of RT-qPCR for all targets was same. Data were analyzed by the 2(-Delta Delta C(T)) method [23].

### Immunoblotting

Total protein was isolated from primary AML cells and cell lines using the RIPA lysis buffer (Beyotime). Protein concentrations were quantified using BCA assay. Equal amount (30 μg) of protein samples from each treatment were separated by SDS-PAGE and then electroblotted onto PVDF membranes (Millipore). Membranes were blocked using 5% nonfat dry milk in TBST for 1 h, and then probed overnight with specific primary antibodies against LC3 (1:1000, Novus Biologicals), ATG7, ATG2B (1:1000, abcam), cleaved caspase 9, pro-caspase 9, cleaved caspase 3, pro-caspase 3 (1:1000, Cell Signaling), BAX and BCL-2 (1: 500, Santa Cruz) and β-actin (1: 5000, Santa Cruz) at 4°C. This procedure was followed by the incubation with HRP-conjugated secondary antibodies (1:10000, Santa Cruz) for 1 h at room temperature. The protein bands were visualized with enhanced chemiluminescence (Thermo Fisher Scientific) and the intensity was analyzed using ImageJ software.

### Luciferase reporter assay

The 3'-UTR of ATG7 and ATG2B was ligated into the firefly luciferase reporter pGL3 vector (Promega). Mutant 3'-UTR of ATG7 (ATG7-mut) and ATG2B (ATG2B-mut) were generated using a QuikChange Site-Directed Mutagenesis kit (Stratagene). HEK293T cells were cultured in 24-well plates and transfected with the 3’-UTR reporter plasmids along with O/E miR-143 or O/E Ctrl. Renilla luciferase expression plasmid was used as control. At 48 h after transfection, cells were harvested and the luciferase activity was measured using a Dual-Luciferase reporter assay system (Promega) following the manufacturer’s instructions. Results are expressed in comparison to O/E Ctrl transfection group.

### Statistical analysis

All data were obtained from at least 3 independent experiments. Data are mean ± SD. Student's *t*-test was applied to compare the data between two experimental groups, unless indicated otherwise. P<0.05 indicates a statistically significant difference.

## Supplementary Material

Supplementary Figures
