# Supplementary material for: MicroRNA-143 sensitizes acute myeloid leukemia cells to cytarabine via targeting ATG7- and ATG2B-dependent autophagy
Source: Aging (Albany NY). 2020 Oct 19;12(20):20111–26. doi: 10.18632/aging.103614 (PMC7655171; doi:10.18632/aging.103614)
Supplement: Supplementary Figures [file aging-12-103614-s001..pdf]

SUPPLEMENTARY FIGURES

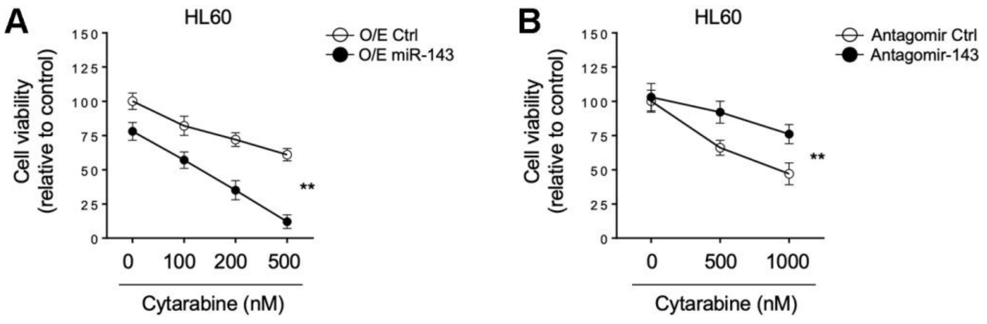

Supplementary Figure 1. miR-143 enhances cytarabine-induced cytotoxicity in AML cells. HL60 cells were transfected with 100 nM miR-143 mimic (O/E miR-143) or 100 nM non-target mimic control (O/E Ctrl) (**A**), or 100 nM antagomir of miR-30a (Antagomir-143) or 100 nM non-target antagomir (Antagomir Ctrl) (**B**) for 48 h. Then, cells were treated with increasing concentrations of cytarabine as indicated for 24 h. Cell viability was analyzed by CCK-8 assay. The results are expressed as relative to vehicle group (%). Each symbol represents the value from 5 replicates. Data were compared using two-way ANOVA with a post hoc Tukey's test. **, P<0.01.

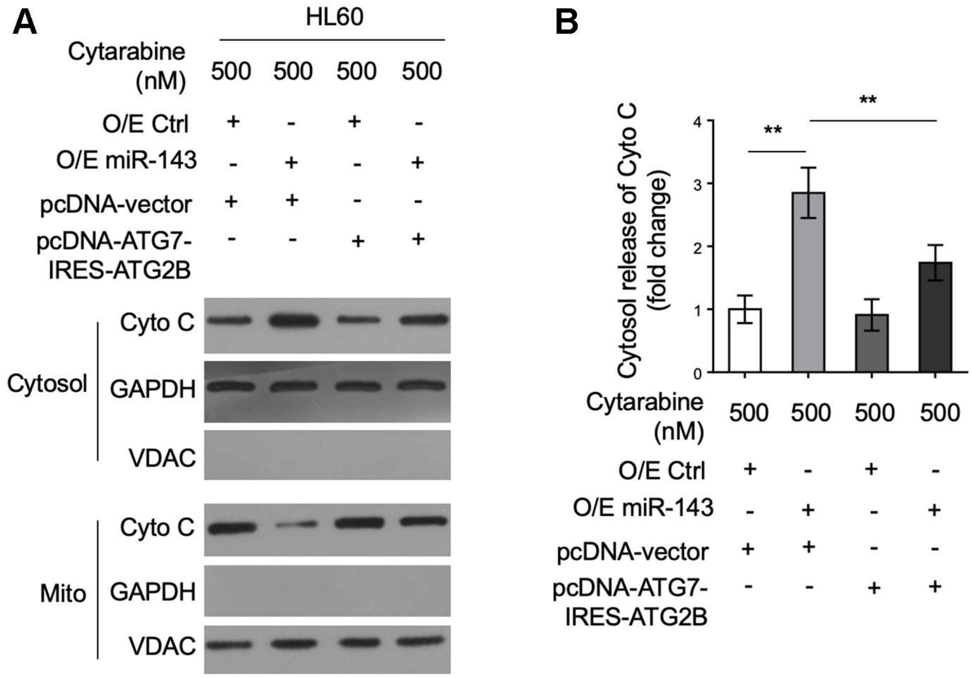

Supplementary Figure 2. miR-143 enhances cytarabine-induced cytosol release of Cyto C in AML cells. HL60 cells were co-transfected 100 nM O/E miR-143 or 100 nM O/E Ctrl with pcDNA-vector or pcDNA-ATG7-IRES-ATG2B for 48 h in the presence of 500 nM cytarabine. The protein expression of Cyto C, and cytosol maker GAPDH and mitochondrial marker, voltage-dependent anion channel 1 (VDAC1), was detected by immunoblotting. The representative images (**A**) and statistical analysis of cytosol release of Cyto C (**B**) are shown.

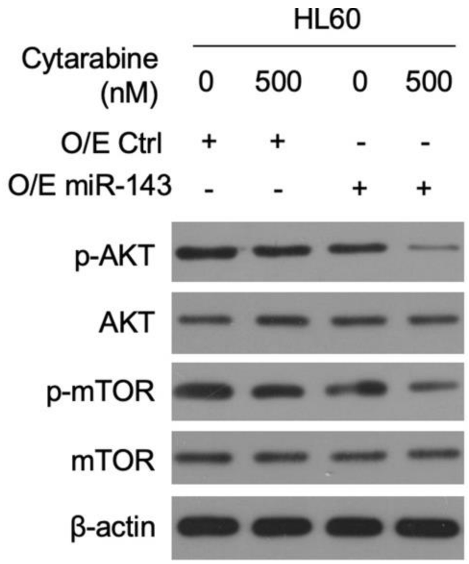

Supplementary Figure 3. miR-143 inhibits Akt/mTOR signaling pathway in cytarabine-treated AML cells. HL60 cells were transfected with 100 nM O/E miR-143 or 100 nM O/E Ctrl for 48 h, and then treated with or without 500 nM cytarabine for 24 h. The protein expression of p-AKT, AKT, p-mTOR and mTOR was measured by immunoblotting. β-actin was used as a loading control. The representative images are shown here.
